# Sampling Considerations for Wastewater Surveillance of Antibiotic Resistance in Fecal Bacteria

**DOI:** 10.3390/ijerph20054555

**Published:** 2023-03-04

**Authors:** Patricia M. C. Huijbers, Julián Bobis Camacho, Marion Hutinel, D. G. Joakim Larsson, Carl-Fredrik Flach

**Affiliations:** 1 Centre for Antibiotic Resistance Research in Gothenburg (CARe), University of Gothenburg, 40530 Gothenburg, Sweden; 2 Institute of Biomedicine, Department of Infectious Diseases, University of Gothenburg, Guldhedsgatan 10A, 40530 Gothenburg, Sweden

**Keywords:** sewage, *Escherichia coli*, hospital effluent, wastewater treatment plant influent, wastewater-based epidemiology, grab sample, composite sample, PhenePlate^TM^, time-kill test, temperature

## Abstract

Wastewaters can be analyzed to generate population-level data for public health surveillance, such as antibiotic resistance monitoring. To provide representative data for the contributing population, bacterial isolates collected from wastewater should originate from different individuals and not be distorted by a selection pressure in the wastewater. Here we use *Escherichia coli* diversity as a proxy for representativeness when comparing grab and composite sampling at a major municipal wastewater treatment plant influent and an untreated hospital effluent in Gothenburg, Sweden. All municipal samples showed high *E. coli* diversity irrespective of the sampling method. In contrast, a marked increase in diversity was seen for composite compared to grab samples from the hospital effluent. Virtual resampling also showed the value of collecting fewer isolates on multiple occasions rather than many isolates from a single sample. Time-kill tests where individual *E. coli* strains were exposed to sterile-filtered hospital wastewater showed rapid killing of antibiotic-susceptible strains and significant selection of multi-resistant strains when incubated at 20 °C, an effect which could be avoided at 4 °C. In conclusion, depending on the wastewater collection site, both sampling method and collection/storage temperature could significantly impact the representativeness of the wastewater sample.

## 1. Introduction

Untreated wastewater can be a valuable source of community-level public health data. Wastewater-based epidemiology has been used to estimate illicit and legal drug use as well as the population prevalence of viruses and bacteria relevant to human infections [1,2,3,4,5,6,7,8,9,10,11]. The main advantage of wastewater analyses over studying individuals is that even a single sample can represent thousands of people, hence allowing population studies with very limited efforts in the actual sample collection. Also, it does not involve acquiring consent from individuals and avoids most of the ethical considerations that relate to generating and handling personal data. For wastewater surveillance of illicit and legal drugs, as well as viruses, the sampling strategy has proven to be important for obtaining samples representative of the population under surveillance [9,10,11,12]. Still, the effect of the sampling strategy on how well the sample represents the bacterial composition in a population remains unclear. Since several studies have recently proposed wastewater monitoring as a promising tool for the surveillance of antibiotic resistance, [1,2,4,13] the need to better understand the effect of the sampling strategy on sample representativeness has become a priority, as highlighted in two recent reviews on wastewater-based epidemiology [14,15].

For wastewater monitoring to correctly reflect the relative abundance of certain traits in bacteria carried by a given human population, wastewater samples must contain bacteria originating from many people without overrepresentation of bacteria stemming from a single or a few individuals. Given that clonality of bacteria is more common within individuals than between individuals, the level of representativeness could be judged by the diversity of strains within a sample, at least for bacteria commonly detected in the human gut flora such as *E. coli*. Simpson’s diversity index (DI) is a measure of diversity that gives particular weight to the evenness of a distribution, in comparison to many other diversity indexes, such as Shannon’s, that give more weight to richness [16]. Indeed, Simpson’s index reflects the probability of two isolates picked randomly from a sample being of the same type and would be heavily affected by the presence of dominant bacterial types. Hence, it would be an informative measure to assess the representativeness of samples for surveillance of antibiotic resistance using wastewater monitoring. However, it requires the characterization/typing of a set of bacterial isolates from each sample to be assessed. While genetic data (particularly whole genome sequencing) provides unmatched resolution, it is a costly and time-consuming approach if many isolates are to be analyzed. Phenotype-based biochemical fingerprinting provides a balance between discriminatory power and the possibility to process many samples simultaneously and has been shown to perform in agreement with genetic fingerprinting methods, such as random amplified polymorphic DNA (RAPD) and enterobacterial repetitive intergenic consensus (ERIC)-PCR, for typing of *E. coli* isolates [17,18]. Studies of *E. coli* diversity using biochemical fingerprinting have presented differences between different types of wastewater samples and suggested that not only sampling location but also sampling method could play a role [19,20,21].

There is also a risk that the representativeness of wastewater samples is impacted during the sampling per se. It was recently shown that sterile-filtered, untreated wastewater from a major Swedish hospital could promote the selection of multi-resistant *E. coli*. [22] The study identified a rapid bactericidal effect on susceptible strains, whereas multi-resistant strains survived and even showed growth in hospital wastewater when incubated at 20 °C. Since wastewater is often collected as 24 h composite samples, this suggests a risk for selection of antibiotic resistant strains in the collection container during sampling. Such selection would lead to a skewed strain composition and a sample not representative of the original sewage. However, it is not known how the observed divergent effect of hospital wastewater on antibiotic resistant and susceptible strains is impacted by temperature.

The overall aim of this study was to provide support for the choice of sampling strategy in future wastewater-based epidemiology studies. Specifically, we aimed to (1) systematically compare different sampling methods (grab and composite) at two different locations (hospital and municipal sites) using *E. coli* diversity as a measure for how well they represent carriage in the human population contributing to the wastewater and (2) investigate how a cooling condition that can be achieved during sampling impacts the differential effect of hospital wastewater on antibiotic-susceptible and multi-resistant strains.

## 2. Material and Methods

### 2.1. Study Sites and Sample Collection

Two sites in Gothenburg, Sweden, were included for collection of untreated wastewater samples. One site (‘municipal’) was at the inlet of an urban wastewater treatment plant (Ryaverket, GRYAAB AB) serving approximately 763,000 individuals. The other site (‘hospital’) was at the main sewer line of one of the Sahlgrenska University Hospital’s main locations, to which the great majority of this hospital location contributes with wastewater. There are approximately 600 staffed beds at this location. It should also be noted that not only patients but also employees and visitors use the toilets at the hospital. The two sites were sampled on seven occasions each between April and October 2018. Wastewater was collected on each occasion and at each site as a single grab sample (‘grab’) and as a seven-hour, composite sample (‘composite’). Composite samples were taken between 8.00 and 15.00 with an automatic sampler of in-house design, which was submerged in the wastewater stream. The sampler consisted of a plastic housing containing a one-liter sampling bottle connected to a pump and a battery-driven steering device (Figure S1). The steering device was set to collect 1 ml of wastewater per 120 s, resulting in approximately 210 subsamples per sampling occasion. Samples were processed within two hours after collection. 

### 2.2. Isolation of E. coli

Samples were shaken vigorously before approximately 25 mL was poured into 50 mL Falcon tubes containing sterile 4 mm glass beads, which were added to improve homogenization. The tubes were vortexed for 30 s and immediately used to prepare serial dilutions of the samples using 0.85% NaCl as diluent. Dilutions were plated on ECC agar (100 µL per plate; CHROMagar, Paris, France) with 3, 15 and 3 plates of the 10^−1^, 10^−2^ and 10^−3^ dilutions, respectively. An increased replication of plates inoculated with the 10^−2^ dilution was included to allow collection of colonies from many separate agar plates. The plates were incubated at 37 °C for 18 h before blue colonies were counted to estimate *E. coli* concentration. Previous studies have shown that *E. coli* can be accurately (>99.5%) distinguished using this sewage cultivation procedure [1,2]. Culture plates were divided into three different pie segments and the two first well-separated blue colonies encountered in each segment, starting from the upper left corner, were picked. To assure non-biased picking of colonies, segments and direction of picking were determined a priori.

### 2.3. Biochemical Fingerprinting of E. coli

In order to estimate diversity, biochemical fingerprints based on the metabolism of eleven carbohydrates and amino acids were determined for 48 presumptive *E. coli* isolates per sample using PhenePlate^TM^-Rapid Screening (PhP-RE) plates according to the manufacturer’s instructions (PhPlate Microplate Techniques AB, Stockholm, Sweden). Absorbance at 620 nm was read after 8, 24 and 48 h of incubation at 37 °C using a microplate reader (FLUOstar^®^ Omega, BMG Labtech, Ortenberg, Germany). After the last reading, the mean absorbance value for each well was calculated and multiplied by ten to yield a biochemical fingerprint consisting of eleven positive integers for each isolate. 

A similarity matrix of correlation coefficients, obtained by pairwise comparison of all biochemical fingerprints, was subjected to cluster analysis using the unweighted pair group method with arithmetic mean. Biochemical fingerprints with correlation coefficients above the specified identity level of 0.975 (software default value) were assigned to the same biochemical type. Diversity was calculated using Simpson’s index of diversity (DI) according to the formula $DI=1-\frac{1}{N\left(N-1\right)}\;\overset{s}{\underset{i=1}{\sum }}n_{i}\left(n_{i}-1\right)$, where *N* is the number of isolates in the sample, *n_i_* is the number of isolates belonging to the biochemical phenotype *i*, and *s* is the total number of biochemical phenotypes in the sample. Values close to one indicate the presence of many distinct biochemical phenotypes evenly distributed, while values close to zero indicate one or more dominant types [23]. Calculations of similarities and Di, as well as cluster analysis, were performed using PhPWIN 7.1 software (PhPlate Microplate Techniques AB). For each run, three control strains were included in duplicates to control for clustering of identical isolates.

### 2.4. Virtual Resampling

To mimic a situation where fewer isolates were collected from several wastewater samples instead of collecting all isolates from a single sample, virtual resampling was conducted. How such sampling affects the diversity of the isolate collections was assessed by comparisons to the original isolate collections. During the virtual resampling, seven new sets of 48 isolates were generated for each type of wastewater sample. The isolates of each new set were evenly distributed between the seven original samples. Each new set was composed of six isolates from one of the original samples and seven isolates from each of the remaining six samples. It was a different original sample that contributed with six isolates in each case. The virtual resampling was performed by simple random sampling without replacement using the strata function of the sampling package in R version 3.6.1 [24].

The diversity of the new sets of *E. coli* isolates was assessed using the previously generated biochemical fingerprints as described above, but this time a lower identity threshold was applied (0.95). This allowed the respective control strains from the different runs to cluster together. The need to decrease the identity threshold when isolates from different PhenePlate^TM^ runs are analyzed together has been described previously [25,26]. 

### 2.5. Time-Kill Test of E. coli Strains in Hospital Wastewater

To examine the killing effect of sterile-filtered hospital wastewater, a time-kill test was performed on a set of antibiotic-susceptible and multi-resistant *E. coli* strains (resistant to at least six of the eleven antibiotics tested). The test was performed according to Kraupner et al. [22] with a few modifications as described below. To examine the possible protective effect of lowered temperature, two different conditions were tested in parallel when the strains were incubated for 24 h in either sterile-filtered hospital wastewater (collected on 7 July 2022) or physiological saline as control. The first, 20 °C at 170 rpm, was used by Kraupner et al. while the second, 4 °C without shaking, was chosen to mimic a condition that can be achieved during wastewater sampling. In addition to the ten strains tested by Kraupner et al. two susceptible (#58 and #102) and two multi-resistant strains (#105 and #127) from the same collection were included (Table S1). Samples, taken at time points 0 h, 5 h and 24 h, were diluted and plated on Mueller-Hinton (MH) agar, which were subsequently incubated at 37 °C overnight before colonies were counted.

### 2.6. Statistical Analyses

Approximate 95% confidence intervals (CIs) for DI were calculated according to Grundmann et al. [27]. Differences in diversity between sampling occasions were assessed for each type of sample (‘hospital, composite’, ‘hospital, grab’, ‘municipal, composite’, ‘municipal, grab’) via overlapping 95% confidence intervals. The Wilcoxon signed-rank test was applied to evaluate differences between sampling methods for each site, whereas the Mann–Whitney test was applied to evaluate differences in growth/survival of antibiotic-susceptible and multi-resistant strains using GraphPad Prism 9.5.0.

## 3. Results and Discussion

### 3.1. Sampling and E. coli Concentration

Composite sample volumes ranged from 128 to 234 mL (median 206 mL) at the municipal wastewater treatment plant and from 109 mL to 214 mL (median 159 mL) at the hospital. The reason behind the lower-than-expected volume in some of the composite samples might be the presence of debris, such as pieces of toilet paper, partly blocking the inlet of the sampling device during parts of the sampling period. However, even in a worst-case scenario, each of the samples were composed of more than 100 subsamples. The concentration of *E. coli* was between 1.4 × 10^4^ and 3.0 × 10^4^ CFU/mL for ‘hospital, composite’ samples; between 2.1 × 10^4^ and 1.4 × 10^5^ CFU/mL for ‘hospital, grab’ samples; between 2.4 × 10^4^ and 7.0 × 10^4^ CFU/mL for ‘municipal, composite’ samples; and between 2.1 × 10^4^ and 7.3 × 10^4^ CFU/mL for ‘municipal, grab’ samples (Table 1). Biochemical phenotypes were determined for a total of 1344 presumptive *E. coli* isolates from 28 wastewater samples. The highest and lowest volumes of collected wastewater did not coincide with the highest and lowest diversities or concentration of *E. coli* in composite samples.

### 3.2. E. coli Diversity in Municipal Wastewater Samples

Diversity was not significantly different between sampling occasions for either ‘municipal, grab’ or ‘municipal, composite’ samples as indicated by overlapping 95% CIs (Table 1). The DIs observed over seven sampling occasions were overall high for both ‘municipal, grab’ (median 0.986) and ‘municipal, composite’ (median 0.978) samples. Wilcoxon-signed rank test showed marginally higher diversity in grab compared to composite samples (*p* = 0.047), which is somewhat counter-intuitive, but might be explained by the fact that composite samples are collected over seven hours where differential survival of strains might affect the *E. coli* composition. Furthermore, even though both types of municipal samples were collected from the same wastewater treatment plant influent (untreated), the grab samples were for technical reasons taken somewhat further downstream within the premises, which possibly could have facilitated further disruption of bacterial aggregates and thus decreased risk of clones in the sample.

The observed high *E. coli* diversity and low variability in municipal influent wastewater across sampling occasions (Table 1), which previously has been reported after composite sampling in other studies, [20,21] suggests that municipal influent wastewater can provide representative community-level data related to *E. coli* carriage, even when a single grab sample is taken. However, if the specific target is a bacterial species or phenotype excreted by few individuals in a community, multiple wastewater samples would still be recommended to increase the chance of capturing such rare bacteria. The number of samples needed would depend on several factors including the rarity of the bacteria and volume wastewater analyzed per sample.

### 3.3. E. coli Diversity in Hospital Wastewater Samples

Compared to the municipal site, the wastewater samples taken at the hospital present a different picture. Three out of seven ‘hospital, grab’ samples and one out of seven ‘hospital, composite’ samples showed significantly lower diversity based on non-overlapping 95% CI’s (Table 1). On those four sampling occasions, between 44% and 67% of isolates belonged to a single common biochemical phenotype. In a previous study at the same hospital, we also observed that single composite wastewater samples can display low *E. coli* diversity due to dominating clones [1] Altogether, it suggests that there is a risk of bacteria from a few individuals being overrepresented in both grab and composite samples. This, in turn, could potentially result in biased community-level data related to *E. coli* carriage due to under- or over-estimation of bacterial traits, such as antimicrobial resistance and virulence, when a single sample of hospital effluent is taken. Low *E. coli* diversity has also been observed when composite wastewater samples (composed of 12–24 subsamples) have been collected at other, similar hospital sites in another part of Sweden and in Norway [20,21] However, in the current study, a Wilcoxon signed-rank test showed that obtained DIs were significantly higher for ‘hospital, composite’ compared to ‘hospital, grab’ samples (*p* = 0.016). The median diversities over seven sampling occasions for grab and composite samples were 0.918 and 0.969, respectively (Table 1). Taking composite samples, and perhaps even multiple composite samples, therefore, appears crucial to obtain representative data when obtaining specimens in similar conditions to those encountered at the hospital site in the current study. Characteristics of this site that distinguish it from the municipal one, and that might contribute to the observed low diversity and high variability, include a smaller contributing population, considerably shorter distance between toilet and sampling point and smaller wastewater volume. All toilets at the hospital are within 500 m from the hospital sampling point, whereas at the municipal sampling point almost no connected toilets are within that distance, and many are more than 20 km away. A shorter distance between toilet and sampling point and smaller wastewater volume implies less homogenization of feces and larger particle sizes, which should increase the risk of sampling several *E. coli* originating from the same individual. Interestingly, when Paulshus et al. analyzed samples collected at a pump station receiving wastewater from a residential area with about 500 inhabitants outside the city of Oslo, they observed lower diversity and higher variability compared to influent samples from the municipal WWTP [21]. This suggests that both sampling method and sampling location should be considered when striving to obtain a sample that is representative of the population under investigation. It also calls for verification of the presence of potential clones when samples are taken in locations where limited numbers of individuals are contributing to the wastewater and the distance between toilet and sampling point is short.

### 3.4. Virtual Resampling

To assess if it would be preferable to collect fewer isolates from several samples rather than the same total number of isolates from a single sample we conducted a virtual resampling for each sample type, where new collections of isolates were composed of an even distribution of isolates from the seven original samples. For the municipal samples, which from the beginning consistently displayed high *E. coli* diversity, the DIs of the resampled collections were similar to the original ones (Table 2). If anything, somewhat lower diversity was observed after resampling, which most likely is a consequence of the lower identity threshold that had to be applied when comparing isolates from different runs. In contrast, the DIs for the resampled collections of hospital isolates were clearly higher compared to the original samples (Table 2). The median diversity for the collections resampled from grab samples was 0.958 and the collections resampled from composite samples showed consistently high diversity with no DI below 0.96. These findings are in line with the observation that clones dominating some of the hospital samples differed between sampling days. The results further suggest that when sampling wastewater from sites similar to the studied hospital, especially when grab sampling is the only option, there is much to gain with regard to diversity by dividing collected isolates between different samples. However, for sites with characteristics like those of the municipal WWTP influent of this study, such a sampling strategy does not seem to be critical for retrieving diverse collections of isolates.

### 3.5. Bactericidal Effect of Hospital Wastewater

**Figure 1:**
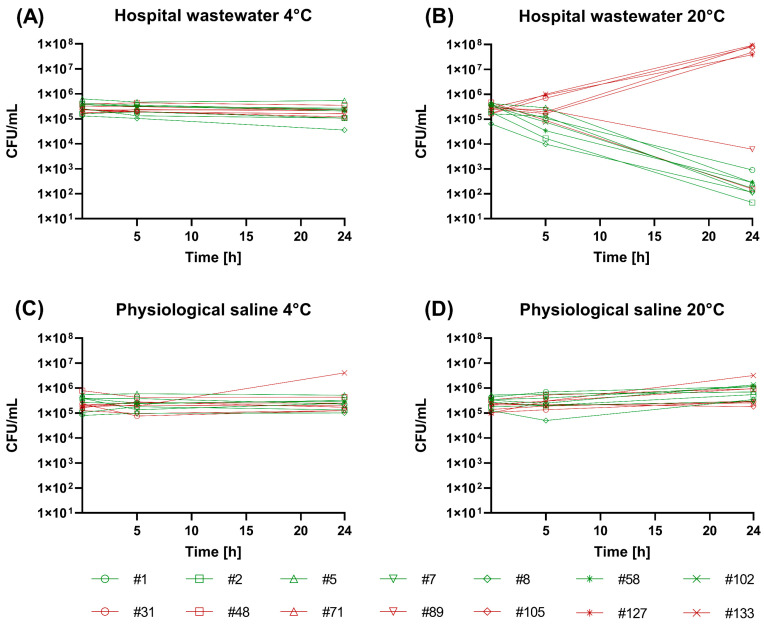
Time-kill assay of antibiotic-susceptible (green) and multi-resistant (red) *E. coli* strains. The individual strains (denoted by different numbers and symbols) were exposed to either hospital wastewater (**A**,**B**) or physiological saline (**C**,**D**) at 4 °C (**A**,**C**), mimicking conditions that can be obtained during wastewater sampling, or at 20 °C (**B**,**D**), identical to the conditions used by Kraupner et al. [22]. CFUs were assessed on MH agar after 5 h and 24 h incubation as well as at the start of the experiment (0 h). The value for strain #89 at 5 h in (**B**) is a minimum assessment since there was overgrowth on the MH plates with the highest inoculated dilution.

The results of a recent study, using wastewater samples from the same collection sites as the current study, suggested a risk of selection of multi-resistant *E. coli* strains during sampling, especially in the case of hospital wastewater [22]. When *E. coli* strains were incubated in sterile-filtered hospital wastewater at 20 °C in the current study, the observed changes in number of CFUs over time confirmed the findings by Kraupner et al. (Figure 1). All the tested susceptible strains showed a marked drop in CFUs after 24 h (median log change −3.18) and were impacted already after 5 h (median −0.64). In contrast, five of the seven multi-resistant strains showed growth, with median log changes of 0.11 and 1.02 after 5 h and 24 h, respectively (Figure 1). The changes in CFUs after exposure to hospital wastewater were normalized to those observed during incubation in physiological saline solution. The normalized CFU changes were significantly different between multi-resistant and susceptible strains, both after 5 h (*p* = 0.042) and 24 h (*p* = 0.018) of incubation at 20 °C as assessed by the Mann–Whitney test.

When the stains were instead exposed to hospital wastewater at 4 °C, no significant differences were observed between susceptible and multi-resistant strains. Both groups of strains could maintain stable CFU numbers (Figure 1), showing median log changes of −0.09 (susceptible strains) and 0.01 (resistant strains), and −0.26 (susceptible strains) and −0.11 (resistant strains) after 5 h and 24 h, respectively. These results advise keeping the collection container cooled during wastewater sampling, to decrease the risk of selection of antibiotic-resistant strains and a skewed strain composition, not least when collecting composite 24 h samples at sites where the chemical content of the wastewater is suspected to exert such a selection pressure. Wastewater from the municipal site of this study has previously been shown to have a small selective effect, if any, for resistant strains [22]. It cannot, however, be excluded that municipal wastewater from parts of the world with higher antibiotic consumption could present a significantly higher risk of selection of resistant strains. 

## 4. Conclusions

This study shows that both the choice of wastewater sampling method and the conditions under which the sample is being collected/stored can, depending on the sampling site, have a significant impact on *E. coli* diversity and strain composition. This, in turn, has implications for wastewater surveillance of antibiotic resistance and, more generally, for any study where bacteria from wastewater are meant to provide information about the microbiota of the contributing population. As wastewater monitoring of bacteria gains popularity as a means to collect health-related data from populations that are otherwise difficult to obtain, [1,2,3,4,13,28,29,30] we would like to highlight the importance of the sampling strategy. For the correct interpretation of the results, it is of the utmost importance that a thorough description of the sampling methods as well as how the collected sample is stored before analysis is provided, and that characteristics of the sampling point and the contributing population are disclosed.

## Figures and Tables

**Table 1:** Wastewater sample characteristics and *E. coli* diversity.

Sample			*E. coli*	Biochemical Phenotypes		Diversity ^c^	95%CI
Type		Date	CFU/mL	Common ^a^	Single ^b^		
Hospital Composite		2018-04-13	3.0 × 10^4^	4	14	0.684	0.532–0.836 ^d^
		2018-06-25	2.2 × 10^4^	8	26	0.979	0.962–0.995
		2018-06-27	1.4 × 10^4^	9	24	0.976	0.957–0.995
		2018-07-05	1.7 × 10^4^	11	16	0.963	0.939–0.987
		2018-10-03	3.3 × 10^4^	9	19	0.958	0.929–0.987
		2018-10-08	2.9 × 10^4^	11	16	0.969	0.954–0.984
		2018-10-17	2.0 × 10^4^	8	23	0.971	0.951–0.991
Hospital Grab		2018-04-13	1.2 × 10^5^	4	9	0.556	0.384–0.728 ^d^
		2018-06-25	4.9 × 10^4^	6	29	0.973	0.948–0.998
		2018-06-27	3.0 × 10^4^	4	27	0.939	0.890–0.988
		2018-07-05	1.0 × 10^5^	4	9	0.741	0.643–0.839 ^d^
		2018-10-03	5.0 × 10^4^	9	8	0.918	0.886–0.950
		2018-10-08	1.4 × 10^5^	3	7	0.658	0.536–0.780 ^d^
		2018-10-17	2.1 × 10^4^	10	18	0.969	0.952–0.986
Municipal Composite		2018-04-18	2.4 × 10^4^	8	25	0.977	0.960–0.994
		2018-06-26	6.9 × 10^4^	7	27	0.978	0.961–0.995
		2018-06-28	7.0 × 10^4^	10	22	0.979	0.965–0.992
		2018-07-04	6.3 × 10^4^	11	18	0.957	0.922–0.993
		2018-10-04	4.7 × 10^4^	7	26	0.961	0.925–0.997
		2018-10-10	4.5 × 10^4^	8	27	0.982	0.968–0.996
		2018-10-16	5.1 × 10^4^	6	32	0.988	0.978–0.998
Municipal Grab		2018-04-19	2.1 × 10^4^	7	31	0.988	0.979–0.997
		2018-06-26	7.2 × 10^4^	5	34	0.986	0.971–1.001
		2018-06-28	7.3 × 10^4^	9	25	0.981	0.968–0.995
		2018-07-04	7.0 × 10^4^	7	28	0.981	0.967–0.995
		2018-10-04	5.2 × 10^4^	7	33	0.992	0.985–0.999
		2018-10-10	3.3 × 10^4^	8	32	0.993	0.987–0.999
		2018-10-16	6.3 × 10^4^	7	29	0.982	0.967–0.997

^a^ Number of types with two or more isolates exhibiting the same biochemical fingerprint. ^b^ Number of types consisting of one unique biochemical fingerprint. ^c^ Simpson’s index of diversity based on biochemical phenotypes of 48 *E. coli* isolates per sample. ^d^ Refers to samples differing from samples not denoted with ^d^ within the same sample type, based on non-overlapping approximate 95% confidence intervals.

**Table 2:** Simpson’s diversity index after virtual resampling in comparison to original sampling.

Sample Type	Simpson’s Diversity IndexMedian (Range)	
	Virtual Resampling	Original Sampling
Hospital Composite	0.969 (0.960–0.979)	0.969 (0.684–0.979)
Hospital Grab	0.958 (0.930–0.962)	0.918 (0.556–0.973)
Municipal Composite	0.972 (0.960–0.983)	0.978 (0.957–0.988)
Municipal Grab	0.976 (0.971–0.980)	(0.981–0.993)

## Data Availability

The data presented in this study are available on request from the corresponding author.

## Supplementary Materials

The following supporting information can be downloaded at: https://www.mdpi.com/article/10.3390/ijerph20054555/s1, Figure S1: Pictures of the automatic sampler used for collecting composite wastewater samples. Table S1: Antibiotic resistance profiles of the *E. coli* strains included in the time-kill test.

## References

[1] Hutinel M., Huijbers P.M.C., Fick J., Åhrén C., Larsson D.G.J., Flach C.-F. (2019). Population-level surveillance of antibiotic resistance in *Escherichia coli* through sewage analysis. Euro. Surveill..

[2] Huijbers P.M., Larsson D.G.J., Flach C.F. (2020). Surveillance of antibiotic resistant *Escherichia coli* in human populations through urban wastewater in ten European countries. Environ. Pollut..

[3] Yan T., O’Brien P., Shelton J.M., Whelen A.C., Pagaling E. (2018). Municipal Wastewater as a Microbial Surveillance Platform for Enteric Diseases: A Case Study for Salmonella and Salmonellosis. Environ. Sci. Technol..

[4] Raven K.E., Ludden C., Gouliouris T., Blane B., Naydenova P., Brown N.M., Parkhill J., Peacock S.J. (2019). Genomic surveillance of *Escherichia coli* in municipal wastewater treatment plants as an indicator of clinically relevant pathogens and their resistance genes. Microb. Genom..

[5] Hovi T., Shulman L.M., Van Der Avoort H., Deshpande J., Roivainen M., De Gourville E.M. (2012). Role of environmental poliovirus surveillance in global polio eradication and beyond. Epidemiol. Infect..

[6] Tiwari A., Lipponen A., Hokajärvi A.-M., Luomala O., Sarekoski A., Rytkönen A., Österlund P., Al-Hello H., Juutinen A., Miettinen I. (2022). Detection and quantification of SARS-CoV-2 RNA in wastewater influent in relation to reported COVID-19 incidence in Finland. Water Res..

[7] Wang H., Churqui M.P., Tunovic T., Enache L., Johansson A., Kärmander A., Nilsson S., Lagging M., Andersson M., Dotevall L. (2022). The amount of SARS-CoV-2 RNA in wastewater relates to the development of the pandemic and its burden on the health system. iScience.

[8] Lindberg R.H., Wennberg P., Johansson M.I., Tysklind M., Andersson B.A. (2005). Screening of human antibiotic substances and determination of weekly mass flows in five sewage treatment plants in Sweden. Environ. Sci. Technol..

[9] Zuccato E., Chiabrando C., Castiglioni S., Bagnati R., Fanelli R. (2008). Estimating community drug abuse by wastewater analysis. Environ. Health Perspect..

[10] Ort C., Lawrence M.G., Reungoat J., Mueller J.F. (2010). Sampling for PPCPs in wastewater systems: Comparison of different sampling modes and optimization strategies. Environ. Sci. Technol..

[11] Thomas K., Bijlsma L., Castiglioni S., Covaci A., Emke E., Grabic R., Hernández F., Karolak S., Kasprzyk-Hordern B., Lindberg R. (2012). Comparing illicit drug use in 19 European cities through sewage analysis. Sci. Total Environ..

[12] Farkas K., Marshall M., Cooper D., McDonald J., Malham S., Peters D., Maloney J., Jones D. (2018). Seasonal and diurnal surveillance of treated and untreated wastewater for human enteric viruses. Environ. Sci. Pollut. Res. Int..

[13] Hendriksen R., Munk P., Njage P., van Bunnik B., McNally L., Lukjancenko O., Röder T., Nieuwenhuijse D., Pedersen S.K., Kjeldgaard J. (2019). Global monitoring of antimicrobial resistance based on metagenomics analyses of urban sewage. Nat. Commun..

[14] Chau K.K., Barker L., Budgell E.P., Vihta K.D., Sims N., Kasprzyk-Hordern B., Harriss E., Crook D.W., Read D.S., Walker A.S. (2022). Systematic review of wastewater surveillance of antimicrobial resistance in human populations. Environ. Int..

[15] Robins K., Leonard A., Farkas K., Graham D., Jones D., Kasprzyk-Hordern B., Bunce J., Grimsley J., Wade M., Zealand A. (2022). Research needs for optimising wastewater-based epidemiology monitoring for public health protection. J. Water Health.

[16] McDonald D.G., Dimmick J. (2003). The concepualization and measurement of diversity. Communic. Res..

[17] Ansaruzzaman M., Albert M.J., Nahar S., Byun R., Katouli M., Kuhn I., Mollby R. (2000). Clonal groups of enteropathogenic *Escherichia coli* isolated in case-control studies of diarrhoea in Bangladesh. J. Med. Microbiol..

[18] Ansaruzzaman M., Bhuiyan N.A., Begum Y.A., Kühn I., Nair G.B., Sack D.A., Svennerholm A.M., Qadri F. (2007). Characterization of enterotoxigenic *Escherichia coli* from diarrhoeal patients in Bangladesh using phenotyping and genetic profiling. J. Med. Microbiol..

[19] Colque Navarro P., Fernandez H., Möllby R., Otth L., Tiodolf M., Wilson M., Kühn I. (2014). Antibiotic resistance in environmental *Escherichia coli*—A simple screening method for simultaneous typing and resistance determination. J. Water Health.

[20] Kwak Y.K., Colque P., Byfors S., Giske C.G., Möllby R., Kühn I. (2015). Surveillance of antimicrobial resistance among *Escherichia coli* in wastewater in Stockholm during 1 year: Does it reflect the resistance trends in the society?. Int. J. Antimicrob. Agents.

[21] Paulshus E., Kühn I., Möllby R., Colque P., O’Sullivan K., Midtvedt T., Lingaas E., Holmstad R., Sørum H. (2019). Diversity and antibiotic resistance among *Escherichia coli* populations in hospital and community wastewater compared to wastewater at the receiving urban treatment plant. Water Res..

[22] Kraupner N., Hutinel M., Schumacher K., Gray D.A., Genheden M., Fick J., Flach C.F., Larsson D.G.J. (2021). Evidence for selection of multi-resistant *E. coli* by hospital effluent. Environ. Int..

[23] Hunter P.R., Gaston M.A. (1988). Numerical index of the discriminatory ability of typing systems: An application of Simpson’s index of diversity. J. Clin. Microbiol..

[24] R Core Team (2019). R: A Language and Environment for Statistical Computing.

[25] Kühn I., Allestam G., Stenström T.A., Möllby R. (1991). Biochemical fingerprinting of water coliform bacteria, a new method for measuring phenotypic diversity and for comparing different bacterial populations. Appl. Environ. Microbiol..

[26] Kühn I., Iversen A., Möllby R. (2003). The PhenePlate system for studies of the diversity of enterococcal populations from the food chain and the environment. Int. J. Food Microbiol..

[27] Grundmann H., Hori S., Tanner G. (2001). Determining confidence intervals when measuring genetic diversity and the discriminatory abilities of typing methods for microorganisms. J. Clin. Microbiol..

[28] Larsson D.G.J., Flach C.F., Laxminarayan R. (2022). Sewage surveillance of antibiotic resistance holds both opportunities and challenges. *Nat. Rev. Microbiol.*
**2022**, 1–2. Nat. Rev. Microbiol..

[29] Newton R.J., McLellan S.L., Dila D.K., Vineis J.H., Morrison H.G., Eren A.M., Sogin M.L. (2015). Sewage reflects the microbiomes of human populations. mBio.

[30] Tiwari A., Kurittu P., Al-Mustapha A.I., Heljanko V., Johansson V., Thakali O., Mishra S.K., Lehto K.M., Lipponen A., Oikarinen S. (2022). Wastewater surveillance of antibiotic-resistant bacterial pathogens: A systematic review. Front. Microbiol..

