# Supplementary material for: Sampling Considerations for Wastewater Surveillance of Antibiotic Resistance in Fecal Bacteria
Source: Int J Environ Res Public Health. 2023 Mar 4;20(5):4555. doi: 10.3390/ijerph20054555 (PMC10002399; doi:10.3390/ijerph20054555)
Supplement: Supplementary file 1 [file ijerph-20-04555-s001.zip › ijerph-2199408-supplementary.pdf]

Supplementary material

Sampling considerations for wastewater surveillance of antibiotic resistance in fecal bacteria

Patricia M.C. Huijbers[1,2], Julián Bobis Camacho[1,2], Marion Hutinel[1,2], D.G. Joakim Larsson[1,2], Carl-Fredrik Flach[1,2,*]

[1] Centre for Antibiotic Resistance Research in Gothenburg (CARe), University of Gothenburg, Gothenburg, Sweden

[2] Department of Infectious Diseases, Institute of Biomedicine, Sahlgrenska Academy, University of Gothenburg, Gothenburg, Sweden

*Corresponding: carl-fredrik.flach@microbio.gu.se

Table S1. Resistance profiles of *E. coli* strains included in the time-kill test.

Strain	MEC	AMC	TZP	CDR	CTX	CAZ	CIP	TOB	NIT	TMP	SXT
#1	S	S	S	S	S	S	S	S	S	S	S
#2	S	S	S	S	S	S	S	S	S	S	S
#5	S	S	S	S	S	S	S	S	S	S	S
#7	S	S	S	S	S	S	S	S	S	S	S
#8	S	S	S	S	S	S	S	S	S	S	S
#58	S	S	S	S	S	S	S	S	S	S	S
#102	S	S	S	S	S	S	S	S	S	S	S
#31	R	R	R	R	R	R	R	R	S	R	R
#48	S	R	S	R	S	R	R	R	S	R	R
#71	S	R	S	R	R	R	R	R	S	S	S
#89	S	R	S	R	R	R	R	S	S	R	R
#105	S	R	S	R	R	R	R	R	S	R	R
#127	S	R	S	R	R	R	R	R	S	R	R
#133	S	R	S	R	R	R	R	R	S	R	R

MEC: mecillinam; AMC: amoxicillin-clavulaninc acid; TZP: piperacillin-tazobactam; CDR: cefadroxil; CAZ: ceftazidime; CTX: cefotaxime; CIP: ciprofloxacin; TOB: tobramycin; NIT: nitrofurantoin; TMP: trimethoprim; SXT: trimethoprim-sulfamethoxazole

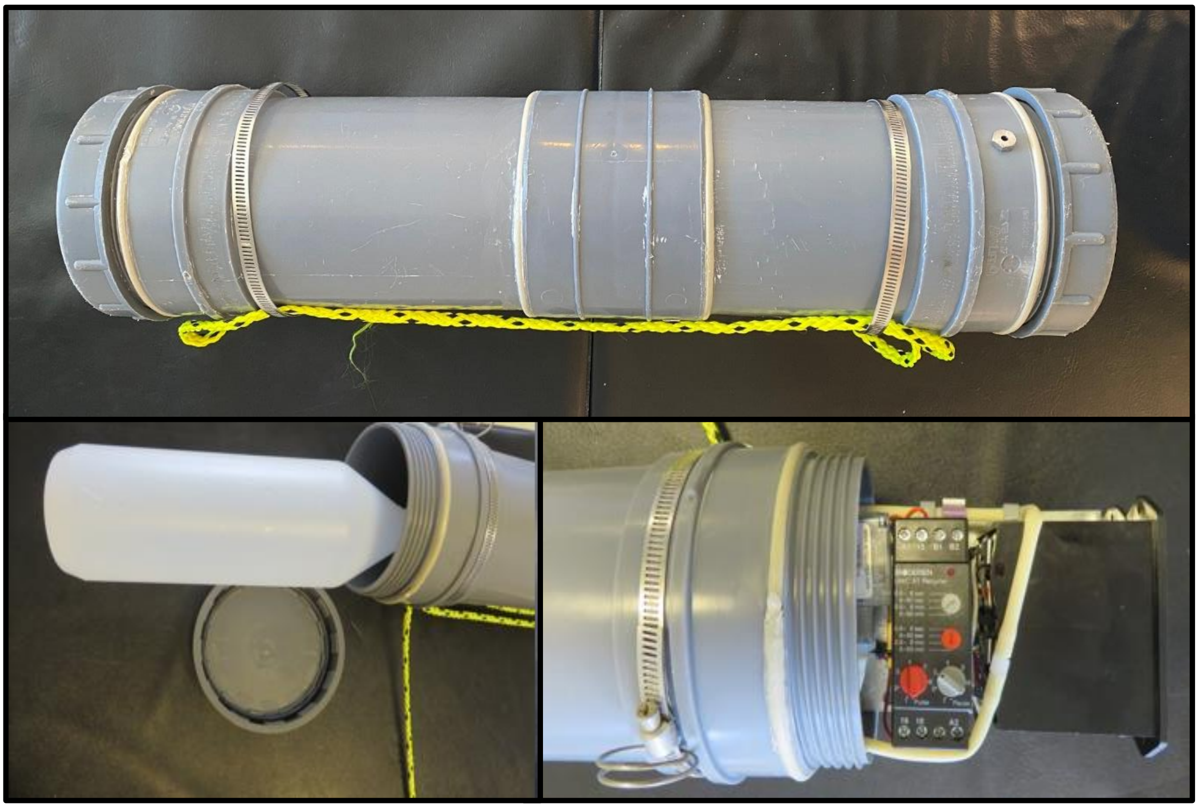

Figure S1. Automatic sampler used to collect composite wastewater samples. The plastic housing of the sampler can be opened in both ends (top). In one end, the one-liter sampling bottle can be reached (bottom left). In the other end, a battery-driven steering devise controlling a pump can be reached (bottom right). The steering devise is used to set the subsample volume and the time interval between the subsamples. Via tubings, the pump is connected to an inlet on the surface of the plastic housing and releases collected volumes in the sample bottle.
